# Barriers and Facilitators to Accessing Preventive Services for Chronic Diseases Among People From Bangladeshi and Nepalese Backgrounds Living in Sydney

**DOI:** 10.1111/hex.70644

**Published:** 2026-03-24

**Authors:** Afsana Anwar, Grish Paudel, Uday Narayan Yadav, Md Nazmul Huda, Abdullah Al Masud, Grace McKeon, Cathy O'Callaghan, Ben Harris‐Roxas, Simon Rosenbaum, Sabuj Kanti Mistry

**Affiliations:** ^1^ Discipline of Psychiatry and Mental Health, School of Clinical Medicine University of New South Wales Sydney Australia; ^2^ School of Population Health University of New South Wales Sydney Australia; ^3^ National Centre for Epidemiology and Population Health The Australian National University Canberra Australia; ^4^ International Centre for Future Health Systems University of New South Wales Sydney Australia; ^5^ ARCED Foundation Dhaka Bangladesh; ^6^ Department of Public Health Daffodil International University Dhaka Bangladesh

**Keywords:** Australia, barriers, chronic diseases, facilitators, people from Bangladeshi and Nepalese origin, preventive care services

## Abstract

**Background:**

People from Bangladeshi and Nepalese origin living in Australia experience a disproportionate burden of chronic diseases, such as diabetes and cardiovascular diseases. Although preventive services are essential to reduce the burden of chronic diseases, existing evidence indicates that these communities encounter unique migration, socioeconomic and health system‐level challenges that impede their access to existing preventive services in Australia. The present study therefore explored the barriers and facilitators to accessing preventive care services among people of Bangladeshi and Nepalese origin living in Sydney, Australia.

**Methods:**

This qualitative study was conducted within a constructivist paradigm, which recognizes that realities are constructed through participants' lived experiences. Six focus group discussions (FGDs) and 22 in‐depth interviews (IDIs) were conducted between August 2024 and January 2025 with people of Bangladeshi and Nepalese origin living in Sydney, Australia. FGDs and IDIs were conducted in participants' native language, transcribed verbatim, translated into English and thematically analysed. The identifed barriers and facilitators to accessing preventive services were organized across multiple levels using the socio‐ecological framework.

**Results:**

Several barriers and facilitators relevant to the contextual experience of people from Bangladeshi and Nepalese backgrounds were identified across multiple levels of the socioecological framework. At the individual level, key barriers included cultural and religious perceptions, limited health literacy and low awareness of available preventive care services. Interpersonal barriers included limited English language proficiency, inadequate availabilty of translated health education materials and interpreter services, and limited cultural understanding among health care providers. Community‐level barriers comprised chronic disease‐related stigma and low level of community engagement. At the institutional and policy levels, barriers included limited culturally tailored support services and infrequent public transport to health care facilities. Conversely, facilitators across these levels included self‐awareness and personal ownership of health, knowledge of available preventive services, peer support network, cultural and linguistic competence of health care providers, the use of digital and social media for health information dissemination, and the supportive role of community organisations.

**Conclusion:**

These findings suggest the need for implementing multi‐level, culturally tailored, community‐led interventions that leverage existing community and social engagement platforms to ensure equitable access to available preventive services for chronic diseases among these disadvantaged population groups in Australia.

**Patient or Public Contribution:**

Study participants contributed to research by sharing their lived experiences of accessing preventive services for chronic diseases. The shared linguistic and cultural backgrounds between the researchers and participants helped rapport‐building and supported in‐depth exploration of the complex factors influencing access to preventive care. Participants provided valuable insights through participating in IDIs or FGDs, which formed the basis of the study findings. However, participants were not directly involved in the study design or conduct of the study, data analysis or interpretation or manuscript preparation.

## 1 Introduction

Chronic diseases, including arthritis, asthma, back pain, cancer, cardiovascular disease, chronic obstructive pulmonary disease and mental health conditions are the leading cause of illness, disability and deaths in Australia [1]. According to the Australian Institute of Health and Welfare (AIHW), 38% of Australians had two or more chronic diseases, and approximately 90% deaths were attributed to chronic conditions in 2022 [1]. Notably, one quarter of the Australian population, who speak a language other than English at home, also known as people from culturally and linguistically diverse (CALD) backgrounds, experience a disproportionate burden of chronic diseases [2].

Bangladeshi and Nepalese communites (originating from two South Asian countries, Bangladesh and Nepal) are two rapidly growing CALD communities in Australia and experience a disproportionately high burden of chronic diseases [2]. According to the 2023 AIHW report, the prevalence of one or more chronic conditions in Bangladeshi and Nepalese origin Australians was 26.9% and 15.2%, respectively [2]. These population groups also reported a higher prevalence of lifestyle‐related chronic diseases such as diabetes and heart diseases [2], for which prevention is critical to reducing chronic disease‐related mortality, morbidit, and disability [3]. Notably, Bangladeshi‐born Australians reported the highest prevalence of diabetes (12%) and heart disease (4.6%) among all population groups in Australia, Nepalese Australians also demonstrated eleveted prevalence of diabetes (6.2%) and heart disease (1.8%) [2]. Despite this eleveted risk profile, there relains limited research that has explored access to preventive care services among Bangladeshi and Nepalese communities living in Australia.

Preventive services for chronic diseases include regular health check‐ups, screening for early detection of chronic diseases such as cancer, diabetes and health promotion activities, including health education sessions and the promotion of healthy diets and physical activity [3]. In Australia, to reduce the burden of chronic disease, the ‘National Strategic Framework for Chronic Disease’ [4] and National Preventive Health Strategy 2021–2030 [5] has been undertaken for the prevention and management of chronic diseases. Other initiatives to support the prevention of chronic disease at the national level include the medicare benefits schedule (MBS), the National Diabetes Services Scheme, and support programs for Aboriginal and Torres Strait Islander peoples with chronic conditions, including the Indigenous Australians' Health Program [4]. A range of comprehensive interventions targeting prevention and management of chronic diseases is also available, for example, early detection programs for cancer and diabetes screening, and lifestyle modification program [4, 6, 7]. Moreover, there is also a dedicated information and support system to manage chronic diseases, for example, health‐direct helpline, cancer learning, better health channel and condition‐specific support groups [6, 7].

Despite the high prevalence of chronic diseases and availability of several preventive care services, multiple studies reported lower utilisation of healthcare services in CALD communities due to several social, cultural and institutional factors [8, 9]. Language barriers and communication challenges, difficulties in adapting to a new culture, limited health literacy, unique socio‐cultural practices, traditions and beliefs of health and well‐being and the complexity of navigating the healthcare system impact access to healthcare services in CALD communities [2, 8, 9, 10, 11, 12, 13]. However, available literature has substantially focused on exploring broader health care access and utilisation among CALD communities, including those of South Asian origin [8, 9, 10, 14, 15], with limited focus on access to preventive services and population‐specific analysis, such as studies focusing specifically on the Bangladeshi or Nepalese populations. For example, Bangladeshi and Nepalese communities might differ from other South Asian communities in terms of language, cultural and religious practices, migration and settlement pathways and experience in engaging health services [12]. All these contextual experiences can shape the perceptions and understandings of chronic diseases and navigating preventive services [12]. Therefore, findings from studies conducted on other or broader South Asian population groups may not be fully transferrable to Bangladeshi or Nepalese‐origin populations. The absence of population‐specific evidence for Bangladeshi and Nepalese populations also limits the development of culturally tailored and equitable preventive care strategies necessary to advance health equity.

Prior research has documented barriers to accessing healthcare services at different levels of health service delivery [8, 9, 10, 14, 15]. This provides important contextual insights across multiple levels of enquiry and highlights the need for multilevel interventions to improve access to care. The socio‐ecological framework is widely used in public health research that enables exploring factors at different levels, that is, health system, organisational, community and individual levels [16]. The model explores factors influencing access to health system, ranging from individual level (e.g. knowledge and attitude) to interpersonal level (e.g. social norms and social network). It also incorporates organisational rules and regulations influencing delivery and uptake of services to the broader community networks, relationship, structures and their impact on accessing services. Further, it considers policy, legislation, advocacy and public awareness that determine the availability and accessibility of the services [16]. The socio‐ecological model was deemed suitable for exploring the barriers and facilitators to access preventive services among Bangladeshi and Nepalese populations, as this model captures the complex interaction of factors such as, individual characteristics, social norms, community behaviours and organisational structure at different levels that determine the access to care. This multi‐level perspective is particularly useful for informing development of interventions that span individual, interpersonal/community, organisational and system levels.

Therefore, the present study aims to identify the barriers and facilitators to accessing preventive care services for chronic disease among Bangladeshi and Nepalese communities in Sydney, using the socio‐ecological framework [16]. By investigating both the barriers and facilitators across multiple socioecological levels, this study seeks to inform the development of culturally sensitive, accessible and equitable preventive care interventions, thereby enhancing the uptake of preventive care services among these populations.

## 2 Method

### 2.1 Study Design

The study followed a cross‐sectional, qualitative research design adopting the constructivist paradigm [17, 18], and the information was collected through in‐depth interviews (IDIs) and focus group discussions (FGDs). Qualitative methods were essential for generating rich insights into motivations, behavioural drivers, structural barriers and broader system‐level influences shaping these behaviours. They are well suited to examining complex interactions between socio‐cultural norms, socio‐economic conditions, community expectations and wider health system contexts [18, 19, 20]. The utilisation of the constructivist paradigm enabled exploration of the barriers and facilitators to accessing preventive care services, delving into the lived experience of the participants [17]. A phenomenological approach was utilised to understand, describe and interpret the perceptions of barriers and facilitators to accessing preventive services, generated through the individual and collective experiences of the participants [21]. Both IDIs and FGDs were conducted to collect complementary information and to examine complex, context‐specific issues, allowing participants to express their experiences freely and flexibly, generating a detailed, nuanced understanding [22].

### 2.2 Study Setting and Participants

This study was carried out in the South‐Western suburbs of Sydney, where the majority of the population are from South Asian backgrounds. The study participants were adults aged 18 years and above from Bangladeshi and Nepalese origin residing in Australia for more than 1 year. The inclusion criteria also required that participants be currently living in Sydney.

### 2.3 Sampling and Recruitment

The research team maintains an ongoing connection with community members from various CALD communities, including Bangladeshi and Nepalese communities in Sydney, through the Multicultural Health Care Support Group (MHCSG) in Sydney. This support group was established by the research team in early 2024, including community advisors from different CALD population groups of Sydney (i.e., Bangladeshi, Nepalese and Indian) to have an ongoing conversation about their health attributes. Recruitment flyers were disseminated by the community advisors of MHCSG to the broader Bangladeshi and Nepalese communities in Sydney through community gatherings, social media and personal networks. MHCSG advisors shared the contact details of potential study participants who verbally agreed to participate and gave consent to share their details with the research team. Members of the research team (A.A., G.P.), contacted the potential participants and checked their eligibility and sent through the participant information statement and consent form via post or email. A total of 6 FGDs (3 from each population group) and 22 IDIs (11 from each population group) were conducted to achieve data saturation, which is aligned with similar qualitative research [23, 24]. Selection of participants also encountered the diversity in terms of age, sex, locality, occupation and type of residency (temporary/permanent) among the Bangladeshi and Nepalese communities living in Sydney [25].

### 2.4 Data Collection

Data were collected through FGDs and IDIs during August 2024 and January 2025, using relevant guides, developed in the English language through an extensive literature review and informal community consultations [15, 23, 26]. These underwent multiple revisions based on the feedback of the research team before finalisation. The final FGD and IDI guides (File S1) covered various domains related to barriers and facilitators to accessing preventive services for chronic diseases. These included participants' understanding of chronic disease and available preventive services, what participant or their families experienced, and what they observed in communities while accessing preventive services. The final FGD and IDI guides were then translated into the Bengali and Nepalese languages for data collection. FGDs were conducted before IDIs to identify a broader perspective before capturing specific nuances, and FGD participants were excluded from IDIs.

All FGDs, each comprising five to eight participants from the same community, were conducted face‐to‐face within the locality of the participants, such as library premises or community park. The FGDs were facilitated in native language of the participants by two research team members (A.A., G.P.), who were PhD candidates with experience in qualitative research and were from Bangladeshi (A.A.) and Nepalese (G.P.) origins. The shared linguistic and cultural background of the researchers allowed building rapport to the study participants and helped exploring in depth social dynamics and complexities within the communities. The lived experiences of the researchers also contributed to gather valuable insights through facilitating community‐embedded collection, analysis and interpretation of the data. In‐language IDIs were conducted by A.A. and G.P. over the telephone based on participants' availability and convenience. The study participants (IDIs and FGDs) received gift vouchers as a token of appreciation for their time and involvement in the study.

### 2.5 Data Analysis

All the FGDs and IDIs were transcribed in Bengali and Nepalese languages and translated into English. The transcription and translation were validated by S.K.M. and U.N.Y who are native speakers of Bengali and Nepalese languages, respectively. We combined both inductive and deductive approaches in analysing the data. While the inductive approach involved open coding across the whole transcripts, the socio‐ecological framework informed the organisation of higher‐level themes. Once preliminary themes were developed, we drew on the socio‐ecological framework to organise and interpret the themes across individual, interpersonal, institutional, community and policy‐level barriers and facilitators to accessing preventive care services for people of Bangladeshi and Nepalese origin living in Sydney [16]. This meant that the coding process itself was inductive, while the socio‐ecological framework informed the subsequent stage of theme categorisation and interpretation, enabling us to situate data‐driven themes within a well‐established framework [16]. By capturing interactions across multiple levels, the socio‐ecological framework supports examination of how social and ecological factors intersect and enables identification of problems across layers of the system. We used NVivo (version 14) for coding and management of qualitative data.

As a first analytical step, two FGD and three IDI transcripts were thoroughly read by two researchers (A.A. and G.P.) to familiarise themselves with the data and to independently develop an initial coding framework. The initial coding frameworks were discussed with the research team for finalisation. Contradictions and inconsistencies were resolved through in‐depth discussion and mutual agreement amongst the research team members. After the emerging codes and categories were finalised, a coding template was developed for the remaining transcripts. Two researchers independently coded the data using the coding template, and 20% of the transcripts were cross‐checked to ensure coding consistency. In the next few steps, codes are collated and grouped into themes, which were then refined through discussion with team members for better presentations and alignment with the socio‐ecological framework [16].

## 3 Results

### 3.1 Characteristics of the Participants

Table 1 outlines the detailed socio‐demographic characteristic of the participants. Participants from both communities varied in terms of age, duration of living in Australia and their residency status. The majority of the participants were female (*n* = 42/63, 66.67%). Most participants had been living in Australia for between 5 and 10 years, with some residing for more than 10 years in both communities, reflecting long‐term settlement experience. The majority of the participants were permanent residents, with asmaller number of temporary residents in both communities provided insight into how residency status may shape engagement with preventive services.

**Table 1:** Socio‐demographic characteristics of the participants.

Community	Variables	Interviews	FGD
**Bangladeshi**	Total number of participants	11 (M‐5, F‐6)	23 (M‐2, F‐21)
	Age distribution of the participants	Between 30 and 60 years, with almost all the participants between 30 and 50 years	Between 25 and 65 years, majority of the participants between 25 and 59 years
	Length in residing in Australia	The length ranges between 1.5 and 17 years. Majority of the participants' residing length was between 5 and 10 years while some of them live for > 10 years and a few for < 5 years	The length ranges between 1 and 30 years. Some of the participants residing for among (1–5) years and (5–10) years duration, with a few of the participants living for ≥ 15 years.
	Residency status:	Majority of the participants were permanent resident	Majority of the participants were permanent residents
Nepalese	Total number of participants in interviews:	11 (M‐8, F‐3)	18 (M‐5, F‐13)
	Age distribution of the participants	Between 25 and 53 years, with majority of the participants between 30 and 40 years	Between 25 and 43 years, majority of the participants aged between 30 and 40 years
	Length in residing in Australia	The length ranges between 4 and 28 years. Majority of the participants' residing length was between 5 and 10 years while some of them live for > 10 years	The residing length between 3 and 17 years, with majority of the participants living for ≥ 10 year, with some of the living between 5 and 10 years
	Residency status:	Majority of them were permanent resident	Majority of them were permanent resident

*Note:* A few refers to < 25%, some refers to (25%–50%), majority refers to (50%–75%) and almost all refers to > 75% of the participants.

**Figure 1:**
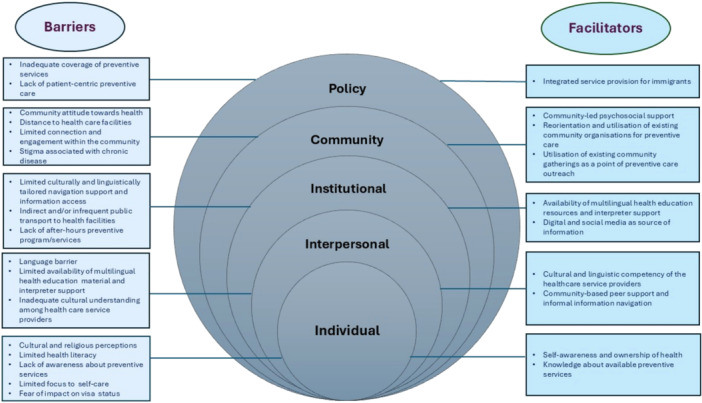
Multiple‐level barriers and facilitators to accessing preventive services among Bangladeshi and Nepalese communities living in Sydney.

We summarised the barriers and facilitators at five broad levels: (1) individual, (2) interpersonal, (3) institutional, (4) community and (5) policy (Figure 1). The broad levels, associated themes and supporting quotes are presented in Files S2 and S3, with representative quotes for specific themes included in the text to enhance understanding.

**Barriers to Accessing Preventive Services for Chronic Disease**

### 3.2 Individual‐Level Barriers

#### 3.2.1 Cultural and Religious Perceptions Related to Chronic Disease

While the participants from Bangladeshi origin framed the chronic illness through religious lenses with cultural‐driven health beliefs and taboos, Nepalese origin participants highlighted the issue of social judgement and boundaries. These distinct cultural and religious perceptions of chronic disease helped to delineate the factors that impeded their access to preventive care. One middle‐aged Bangladeshi male participant who is an Australian citizen quoted that:

Especially many of us think that diabetes is God given. I have nothing to do here. But many people don't think that it is a modifiable matter, within a person's control. So, according to religious faith, it is considered pre‐destination by all.

(BD‐FGD2‐P3)

A Nepalese female participant who is a permanent resident in Australia quoted that:

Sometimes, cultural background and strict rules create certain boundaries. For instance, some people feel uncomfortable opening to doctors, which can be a significant issue. This reluctance to speak openly about health concerns can prevent individuals from seeking help or accessing necessary healthcare services.

(Nepalese‐FGD2‐P1)

#### 3.2.2 Limited Health Literacy About Chronic Disease

Limited health literacy about chronic disease was identified as another barrier. Many participants reported being unaware of the definition and types of common chronic diseases. One of the interview participants from Bangladeshi origin (M, age 40), who is an Australian citizen living in Sydney for 8 years said:

I googled the chronic disease today at the time of interviewing you. I thought chronic disease meant cancer or something big like that. But I did not know that diabetes or hypertension are chronic diseases.

(BD‐IDI‐3)

Participants observed that many individuals within their communities have limited understanding of their chronic conditions, even after receiving a formal diagnisis.

I believe poor health literacy is one of the barriers. Even when people are diagnosed with chronic conditions, they know little about their health problems.

(Nepalese‐IDI‐2)

#### 3.2.3 Lack of Awareness About Preventive Services

Participants from Nepalese origin frequently identified the lack of awareness about available preventive services as a hindrance to accessing them. Participants mentioned a lack of information about what preventive care services cover and what services are offered by the health system. One Nepalese male participant living in Syndey on temporary visa quoted as following:

No, we have not accessed…. We don't know what services are available here in Australia or what the government offers.

(Nepalese‐IDI‐9)

#### 3.2.4 Limited Focus to Self‐Care

Limited focus on self‐care was identified as a barrier to accessing preventive services, which was frequently reported in FGDs by participants from both communities. Competing prioritising such as work commitments, financial instability, pressure of balancing work and personal life, and caregiving responsibilities often deter individuals from prioritising their health, leading to low engagement with preventive services.

Often, their priorities are focused more on work and financial stability than on health, which leads to low engagement in preventive healthcare.

(Nepalese‐FGD2‐P1)

Many Bangladeshi participants pointed out that women tend to neglect their health because of their daily caregiving responsibilities for their families. One of the interview participants of Bangladeshi origin (female, age 50), who is an Australian citizen and living in Sydney for 15 years, quoted as follows:

They neglect their health. Women are special. They completely ignore their health. They come here and become so busy with their husband's career, job, and their children's schoo. They do not exist on their own. As a result, various diseases occur. Diabetes is there, along with many other complex chronic diseases. Many also develop mental health‐related problems.

(BD‐IDI‐5)

This negligence complemented with their lack of self‐confidence and self‐care, which deteriorates their physical and mental health, delaying access to preventive care.

#### 3.2.5 Fear of Impact on Visa Status

Fear of a negative impact on visa status, including potential implications for obtaining permanent residency, emerged as a significant barrier to accessing preventive health care services among both Bangladeshi and Nepalese participants.

I have seen closely, one of my friends, who didn't have permanent residency at the time, may have felt restricted in accessing healthcare services here.

(Nepalese‐FGD2‐P3)

### 3.3 Interpersonal Level Barriers

#### 3.3.1 Language Barrier

Limited English language proficiency emerged as one of the significant barriers to accessing preventive care services among both Bangladeshi and Nepalese communities. Participants from Nepalese origin described how overall language difficulties hindered not only their verbal communication but also access to free‐of‐cost services. In contrast, participants of Bangladeshi origin described unfamiliar dialects and accents of the health service providers as a key communication challenge. One Nepalese female permanent resident desribed how language difficulties hinder service uptake, stating:

Even the free‐of‐cost services that the government offers through community services often go untapped within the Nepali community. Whether knowingly or unknowingly, the language barrier prevents people from fully accessing these services.

(Nepalese‐FGD2‐P1)

One Bangladeshi female participant, who currently works as a health worker, described challenges related to unfamiliar dialect and accent as follows:

I didn't even understand their accent. Canterbury Hospital had an Indian Bengali‐speaking interpreter in the antenatal sector; she explained everything to me. But unfortunately, I could not catch the classes on diabetes for even one day.

(BD‐IDI‐5)

#### 3.3.2 Limited Availability of Multilingual Health Education Material and Interpreter Support

Participants of Bangladeshi origin highlighted the absence of health‐related education materials translated into Bengali and the limited availability of interpreters, which limited access to health information and related services. A Bangladeshi‐origin older adult Australian citizen (female, age 65) described her expereince as follows:

There is an interpreter, but there is no Bengali version for various documents. Many leaflets, but if they are in Bengali, people can read and understand. A lot of information can be found.

(BD‐FGD2‐P1)

#### 3.3.3 Inadequate Cultural Understanding Among Health Care Service Providers

Although many participants from both Bangladeshi and Nepalese backgrounds described positive interaction with health service providers, some Nepalese participants reported a lack of understanding among healthcare providers regarding their health beliefs, culture and traditions.

I also feel that GPs do not want to understand or assess our health beliefs, culture, and traditions.

(Neplese‐IDI‐3)

### 3.4 Institutional‐Level Barriers

#### 3.4.1 Limited Culturally and Linguistically Tailored Navigation Support and Information Access

Participants reported that limited culturally and linguistically tailored navigation support, along with insufficient information about the health care system and available preventive care services, affected people's ability to seek preventive care services in a timely manner. One Nepalese permanent resident female participant quoted:

There's a lack of information and navigating the healthcare system can be overwhelming without proper guidance or awareness. This leads to many people missing out on preventive care and timely treatment.

(Nepalese‐FGD3‐P5)

#### 3.4.2 Indirect and/or Infrequent Public Transport to Health Facilities

Participants of Bangladeshi origin emphasised that the absence of direct and/or infrequent public transport services to health care facilities acted as a barrier to accessing preventive health care, particularly for individuals who rely on public transport or do not have private vehicles.

Like Canterbury Hospital, it is near, not too far. But leaving Lakemba, I think, becomes difficult. Because I must come to the station from my house, from here again go to Canterbury by train, then go again by bus.

(BD‐FGD1‐P6)

#### 3.4.3 Lack of After‐Hours Preventive Program/Services

Participants reported that limited after‐hours preventive services was identified as a barrier to accessing preventive care services, particularly for individuals whose work commitments did not allow attendance during working hours. One Bangladeshi participant (female, age 43) who has been living in Australia for 17 years, stated:

Various awareness sessions are held for diabetes. I do participate in those awareness sessions. But he doesn't have time as he has work at that time and the sessions are in working hours.

(BD‐IDI‐9)

### 3.5 Community‐Level Barriers

#### 3.5.1 Community Attitude Towards Health

Participants from both communities reported that negative community attitudes towards health was a significant barrier to accessing preventive health care services among Bangladeshi and Nepalese communities. Delayed health‐seeking behaviour, reluctance to consult health professionals, and seeking care only during emergencies were reported to hinder access to preventive health services.

Overall, Nepalese‐origin individuals tend to neglect their health, skipping routine checkups and only seeking care during emergencies or severe illnesses.

(Nepalese‐IDI‐5)

#### 3.5.2 Distance to Health Care Facilities

Long distance to health care facilities were identified as a significant barrier to accessing preventive care services.

Distance to healthcare facilities also plays a role in seeking care. In the past, I had to travel a long distance for counselling, which was difficult for me.

(Nepalese‐IDI‐2)

#### 3.5.3 Limited Connection and Engagement Within the Community

Participants from both communities reported limited social connection and engagement within the community as a barrier to accessing preventive care services. Bangladeshi participants described an increasingly individualised lifestyle, whereas Nepalese participants emphasised limited social networks and community involvement, which shaped information pathways within their communities.

Here's what I see, you just be yourself, no one tells you anything. Everyone is busy with themselves.

(BD‐IDI‐1)

Additionally, social interactions and community involvement tend to be minimal…. Participation in community and volunteering activities is quite low, further limiting opportunities for social connection and engagement.

(Nepalese‐FGD2‐P1)

#### 3.5.4 Stigma Associated With Chronic Disease

Chronic disease‐related stigma emerged as a significant community‐level barrier to accessing preventive health care services in both Bangladeshi and Nepalese communities. Bangladeshi participants described personal discomfort in sharing health‐related information, while Nepalese participants expressed their concerns about fear of judgement, shame and social isolation, which hindered timely support‐seeking and access to preventive care services. These different layers of stigma shaped how the people engage with preventive cares.

It's a big barrier to going to the doctor, plus a barrier to sharing information…. It may take them a long time to think about sharing this topic. This attitude is strong among Bangladeshis.

(BD‐IDI‐5)

……. but social stigma plays a significant role in the Nepalese community. Many individuals with mental health conditions prefer to remain unidentified and isolate themselves due to fear of being judged by their families, relatives, or community. This hesitation prevents them from seeking timely medical support.

(Nepalese‐IDI‐6)

### 3.6 Policy‐Level Barriers

#### 3.6.1 Inadequate Coverage of Preventive Services

Participants from both communities reported that limited accessibility, visibility, and cultural relevance of preventive services, along with inconsistencies across geographical locations, constituted barrier to accessing preventive care services. For example, one Bangladeshi FGD participant (male, age 57), currently an Australian citizen, stated:

These programs are not visible. Not particularly visible in community settings, and they lack accessibility. The main reason for the lack of accessibility is that they are not exposed. and not culturally acceptable at the community level.

(BD‐FGD2‐P3)

#### 3.6.2 Lack of Patient‐Centric Preventive Care

Participants expressed concerns that services were predominately focused on advocacy and operated through a top‐down approach, rather than actively targeting and engaging community members most in need. This highlights a disconnect between the current advocacy framework, existing service delivery models, and tangible benefits for communities.

These programs are not just patient‐centred services in New South Wales; they are more advocates. They do more advocacy, but they don't do much for the common people.

(BD‐FGD2‐P3)

**Facilitators for accessing preventive services for chronic disease**

### 3.7 Individual‐Level Facilitators

#### 3.7.1 Self‐Awareness and Ownership of Health

Self‐awareness and a sense of ownership over health were identified as facilitators of preventive care access. Some participants descibed how improved self‐awareness of chronic disease motivated them to engage in preventive activities.

If my diabetes goes above 9, I feel very sick. I see many going on 12/13, but I can't. My body tells me that my diabetes has increased, so I am forced to do so much. That's why I am so aware.

(BD‐IDI‐6)

#### 3.7.2 Knowledge About Available Preventive Services

Knowledge of available preventive services emmerged as another important facilitator of preventive care access. Several participants reported that awareness of available preventive services increased their intention to utilise them.

As far as I know Metro Assist or the library sometimes conducts some sessions of yoga exercise, chair sitting type of activities for the elderly, long‐term spine problems, diabetes problems……Chair sitting activity, morning walk activity, these I have seen on behalf of Metro Assist, plus the initiatives taken by Lakemba Library in the community.

(BD‐FGD3‐P4)

### 3.8 Interpersonal Level Facilitators

#### 3.8.1 Cultural and Linguistic Competency of the Healthcare Service Providers

Sharing a similar cultural and linguistic background with healthcare providers was identified as a facilitator, as such similarity eased communication between providers and service users.

I find it easier to communicate with Nepalese GPs as we share the same language and cultural background.

(Nepalese‐IDI‐1)

#### 3.8.2 Community‐Based Peer‐Support and Informal Information Navigation

Informal peer support groups and information sharing were identified as additional interpersonal‐level facilitators across several IDIs and FGDs in both communities. Participants described how casual social gatherings and group activities facilitated shared experience and exchange of information.

Social gatherings where people talk openly about chronic disease. There is a group of people who are diabetic. They will walk or do some activity together…then communication will be fine.

(BD‐IDI‐7)

Participants further reported utilising informal peer groups, including family members, friends and peers, as a primary sources of information about preventive services.

When someone in my family has a chronic condition, I usually seek advice from other senior Nepalese people or contact the hospital for information. This is what my family and I typically do.

(Nepalese‐FGD‐P4)

### 3.9 Institutional‐Level Facilitators

#### 3.9.1 Availability of Multilingual Health Education Resources and Interpreter Services

Participants reported that the availability of translated chronic disease‐related education materials and interpreter services could facilitate access to the preventive services, and recommended extending these services to primary care settings to support effective communication.

There is an interpreter, but there is no Bengali for various documents. Many leaflets, but if they are in Bengali, people can read and understand. A lot of information can be found.

(BD‐FGD2‐P1)

#### 3.9.2 Digital and Social Media As Source of Information

Participants highlighted the use of existing digital and social media platforms to disseminate information and increasing awareness on chronic disease‐related preventive services due to their broad reach.

I believe social media is the most practical solution for spreading information. By creating dedicated health‐focused pages, we can share valuable information, tips, and resources directly with the community.

(Nepalese‐FGD‐P3)

Participants further reported that mobile SMS messaging, already used participants for disseminating information about various services, could be an effective way to reach individuals with limited access to or knowledge of internet use.

I think because everyone uses mobile phones….……. …. For them mobile SMS can be given in different languages, it can be helpful to pass information like we get for different things.

(BD‐FGD3‐P4)

Participants also identified radio program as an effective communication channel for raising awareness of preventive health services and available healthcare programs.

Radio ads could be an effective way to raise awareness about preventive health services and available healthcare programs……. who may not actively seek out information online or through pamphlets.

(Nepalese‐FGD‐P6)

### 3.10 Community‐Level Facilitators

#### 3.10.1 Community‐Led Psychosocial Support

Community‐led psychosocial support emerged as a facilitator for coping with stigma, isolation and psychological stress, thereby enhancing access to the preventive care services.

Bangladeshis can give each other mental support. They can help in different ways. The help that I get. Many Bangladeshi brothers whom I don't even know visit me.

(BD‐IDI‐11)

#### 3.10.2 Reorientation and Utilisation of Existing Community Organisations for Preventive Care

Many Nepalese participants reported that some community organisations primarily focused on fundraising for treatmentrelated support. Participants emphasised the need to reorient these organisations towards facilitatating access to preventive care service through education and navigation support.

However, I believe these organizations tend to be more reactive than proactive. There is a need for them to focus more on raising awareness and actively advocating for health issues at the community level, rather than only responding to situations as they arise.

(Nepalese‐FGD2‐P3)

#### 3.10.3 Utilisation of Existing Community Gatherings As a Point of Preventive Care Outreach

Participants identified community festivals, gatherings and community spaces (e.g. schools, parks) as key touchpoints for disseminating preventive care services and improving access.

The Bangladeshi community has many festivals. There are various health promotion programs in these festivals, those who work with the community can provide booths. They can do community consultation within the booth. If you go to these places, you will get this service.

(BD‐FGD2‐P3)

### 3.11 Policy‐Level Facilitators

#### 3.11.1 Integrated Service Provision for Immigrants

Some participants highlighted the importance of integrating preventive services with existing social support services, such as Centrelink and MediCare to promote better access to care.

Immigrants face multiple challenges as they move from one country to another. So, here, comprehensive patient‐centred needs for immigrants need to be understood by the government. And these should be tagged with support services like Centrelink, MediCare.

(BD‐FGD1‐P3)

## 4 Discussion

This study provides one of the first population‐specific, preventive care‐focused insights into the barriers and facilitators influencing access to preventive care services among Bangladeshi and Nepalese communities living in Sydney. By applying the socio‐ecological framework, the study identified multi‐level factors that shaped preventive care engagement among these two underserved CALD communities in Sydney. These findings contribute valuable evidence to address existing knowledge gaps and informed the development of culturally responsive, equity‐oriented policy and practice reforms to enhance access to preventive care services.

### 4.1 Barriers to Accessing Preventive Care Services for Chronic Diseases

Cultural and religious perceptions of chronic disease, limited health literacy and awareness about chronic diseases and limited focus on self‐care emerged as key individual‐level barriers to accessing preventive services, consistent with findings of prior literature. Studies conducted among South Asian and other CALD communities living in the United Kingdom, Canada, the United States and Australia have consistently reported challenges in navigating healthcare system due to limited health literacy [27, 28]. A previous study that applied the socio‐ecological framework to examine participation to cervical cancer screening among Pakistani and Somali immigrant women and found that limited health literacy hindered access to screening services [29]. Similarly, a lack of awareness about available preventive services has been shown to limit access to those services among South Asian migrants [9]. Moreover, aligned with prior studies conducted among South Asian migrants in Australia [11, 30], the present study also reported that work pressure, financial instability and caregiving responsibilities often result in undermining self‐care among the participants, limiting their engagement with preventive care. These findings are consistent with findings from previous studies conducted among South Asian communities in Australia [11, 30]. Although these findings align with the broader migrant health literature, this study highlights the distincts ways in which Bangladeshi and Nepalese communities perceive and understand chronic illness. Although barriers such as cultural and religious beliefs, limited health care knowledge, and lack of awareness of existing healthcare services have been documented among other South Asian communities in Australia, this study reveals a distinct concern among Bangladeshi and Nepalese participants regarding the perceived impact of preventive care utilisation on visa status. Many partiicpants feared that being diagnosed with chronic conditions could compromise their chance of obtaining permanent residency, which was especially pronounced among those who were on different kinds of temporary visa. These findings highlight the need for culturally appropriate health education initiatives to improve health literacy and raise awareness on available health services and eligibility of people accessing them [31].

At the interpersonal level, limited English language proficiency and inadequate availability of multilingual health education materials and interpreter services were the key barriers to accessing preventive care services. Although these barriers were similar in both communities, their way of experience was different. Bangladeshi participants described the accent and dialect‐related difficulties, limited availability of translated materials into Bengali and inadequate interpreter support, whreas Nepalese participants reported overall language barriers. Limited English language proficiency hinder effective communication, understanding materials written in English and navigation of complex health system, particularly for individuals whose first language is not English [32]. This finding aligns with previous research on South Asian migrants and CALD communities in Australia and other countries [2, 9, 11, 29, 30]. Although interpreter services and translated health education materials are available in various languages [33], this is limited for Bengali and Nepalese people in Australia. Community outreach activities and educational programs that incorporate bilingual community health navigators, onsite interpreter services and translated education materials in native languages could play an effective role in improving access to preventive care for Bnagladeshi and Nepalese communities [30, 31, 34]. Moreover, lack of cultural understanding among health care service providers was identified as a barrier to accessing preventive care services, aligned with previous studies [8, 15, 34]. The development of a culturally competent, empathetic framework [35], placing importance on providing care in a culturally appropriate manner is required. Training on culturally responsive service delivery [15, 32] can also result in improved cultural responsiveness amongst health service providers.

Participants expressed challenges in accessing preventive care services due to limited availability of culturally tailored health information and navigation services. This finding is consistent with previous studies conducted among CALD communities in Australia [15, 36]. Utilisation of bilingual community health navigators and the provision of health education materials in native languages can be effective in alleviating these barriers. Inequitable public transport infrastructure and unavailability of after‐hours preventive care services were also identified as institutional‐level barriers to accessing preventive services. Unavailability of adequate public transports has often been cited as a major barrier impacting access to healthcare [37]. These urges reorganising the public transport infrastructure in collaboration with health policy makers, urban planners and transport experts [37]. Unavailability of after‐hours preventive care service limits people's ability to access the preventive services, which is particularly critical for individuals who have long working hours and are burdened with different household and care responsibilities. Although some available after‐hours support is available for primary and emergency care in Australia [38], preventive care services for chronic diseases remain limited within the health system. The implementation of after‐hours preventive service delivery model [39] could strengthen health system responsiveness and improve equitable access to preventive care.

A negligent community attitude towards health emerged as a significant community‐level barrier to promoting access to preventive services. Delayed health‐seeking behaviour, perceptions that occasional visit to health professionals are sufficient for managing chronic diseases, and reliance of healhcare services only during an emergency were identified as barrriers to accessing preventive care services. This behaviour aligns with chronic health‐related attitude observed among South Asian poualiton, which tend to prioritise curative care over preventive approach [40]. Alongside this, limited social connection and engagement within the community has been identified as a barriers to accessing preventive care services. Prior research also documented that strong community support and social network results in better participation to preventive services [41]. An individualistic lifestyle and work‐related pressures lead to limited community participation, reduced interpersonal communication, and increased social isolation, which can contribute to reduced information flow and underutilisation of preventive services [30, 36]. Community‐wide stigma related to chronic disease is reported to be a critical barrier for accessing preventive services by majority of the participants. Fear of judgement, shame and hesitancy to share health‐related information is found in the South Asian communities. This may lead to delayed seeking and accessing preventive services [42]. Moreover, engagement with preventive services might be considered as a ‘sign of weakness’, hindering people's access to preventive services in CALD communities [43]. Community‐based and culturally appropriate health education programs may help to improve community attitudes towards preventive care and reduce stigma [31]. The formation of peer support groups can be an effective strategy for strengthening community engagement and social support, thereby improving access to preventive care services [44]. Moreover, long distances to health care facilities was also identified as a community‐level barrier to access preventive services. Long travel distances to health care facilities, coupled with inadequate public transport system and reliance on private vehicles influence people's health care access behaviour [45]. Limited English language proficiency and poor communication skills adds an additional a layer of complexity in finding existing facilities and arranging transport, while transport cost acts as a barrier for CALD communities, especially for those who rely on public transport [15]. Transportation incentives, including bus passes, taxi vouchers and free or reimbursed transportation costs, can help improving access to preventive services [46].

Participants expressed dissatisfaction with current preventive care services for their inadequate coverage and lack of a patient‐centric approach. Although CALD communities are recognised as a priority population for chronic disease prevention in Australia, the health system has not fully met the needs of Bangladeshi and Nepalese communities. This gap highlights the need to develop culturally tailored preventive care service models for chronic disease that meaningfully incorporate the needs, perspectives and voice of the population from Bangladeshi and Nepalese origin.

All the barriers that were identified at the different levels play an interconnected role in shaping the preventive care access by Bangladeshi and Nepalese communities living in Sydney. Limited health literacy, awareness and self‐focus together with limited availability of interpreter service and multilingual health education material and cultural and religious perceptions limit the accessibility of available materials and services, complicate the health service navigation and deject the regular health check‐up and engagement with other preventive services. Visa‐related fear also plays a negative role in preventive service accessibility, especially those who are on different temporary visas and prioritise their visa security over health. All the barriers identified thus contribute to delayed engagement with preventive care services including regular screening, lifestyle modification and early detection of chronic disease and may lead to delayed diagnosis and adverse outcomes related to chronic disease. Identified barriers thus downstream the health outcomes in Bangladeshi and Nepalese communities.

### 4.2 Facilitators for Accessing Preventive Care Services for Chronic Disease

Participants also noted several facilitators to accessing preventive services across all levels of the socioecological framework. Community‐based peer support and informal navigation, reorientation and utilisation of existing community organisation and digital and social media as a source of information were identified as most prominent facilitators.

Some participants identified individual self‐awareness, ownership of personal health and adequate knowledge about available preventive services as facilitators to accessing preventive care services. Prior research also documents that providing importance to one's own health and having information on available services improve access to care [32, 47].

Cultural and linguistic competency of healthcare providers was identified as a key interpersonal‐level facilitator. This finding aligns with studies conducted in South and Southeast Asian immigrants in Japan [48] and Pakistani and Somali immigrant women in Oslo [29]. Both studies used the socio‐ecological framework and reported that cultural and linguistic competency of health service providers helps to address language barrier and improve communication to facilitate utilisation of preventive services [30]. Peer support and informal navigation can serve as a trusted way of sharing information and promoting behaviour change [44].

At the institutional level, availability of multilingual education resources and utilisation of digital and social media as information source were identified as facilitators for accessing preventive services. These findings are aligned with previous studies conducted on South and Southeast Asian migrants [48] and Pakistani and Somali immigrants [29]. Availability of multilingual education resources facilitates the dissemination of information through trusted sources and organisations [48] and can facilitate better access to care.

Availability of community‐led psychological support was identified as a facilitator for accessing preventive services. Psychological distress can hinder the utilisation of preventive care services [49], and psychological support from community members can improve access to preventive care. Participants from Nepalese community critiqued the fact that community organisations were not focused on chronic disease prevention and suggested reorientation of community organisations to promote access to preventive care among community members. This is aligned with the findings of a qualitative study which explored the role of community organisations in improving access to community resources for chronic disease prevention and management [50]. Most participants emphasised the use of social gatherings as an effective platform to promote the importance of accessing preventive services. Participants advocated for the integration of chronic disease prevention services with other social support services such as Centrelink and Medicare. A comparable model in Canada, Accessing Canadian Healthcare for Immigrants: Empowerment, Voice & Enablement (ACHIEVE) program, has been proven successful in improving health care utilisation among immigrants [47]. Adaptation of this kind of immigrant‐focused program within the Australian context might be beneficial for underexplored communities, such as Bangladeshi and Nepalese populations.

The findings of the research have significant implications for policy and practice in improving access and utilisation of preventive care services among Bangladeshi and Nepalese populations in Australia. Aligned with the findings of this study and the preventive healthcare strategy (2021–2032) [5], health services should prioritise culturally sensitive health promotion interventions that provide clear information on available preventive care services, effective navigation pathways to those services, eligibility to participation in relation to visa conditions, and access to multilingual health education materials and services. Health services should also consider developing culturally tailored health promotion and navigation interventions across multiple levels of health service delivery (e.g. individual, community, organisation) to promote better access to preventive care services among Bangladeshi and Nepalese communites. It is also important that health care delivery is culturally appropriate and that health services consider population‐specific needs, such as availability of multilingual healthcare materials and interpreter services while delivering preventive care services for specific population groups experiencing disproportionate chronic disease burden.

### 4.3 Strengths and Limitations of the Study

This study is among the first providing insights into the barriers and facilitators to accessing chronic disease preventive services among people of Bangladeshi and Nepalese origin living in Australia. The use of both FGDs and IDIs enabled in‐depth exploration of perspectives from both communities regarding barriers to accessing preventive care. A key strength of the research was the established relationship between the research team and community representatives, which ensured trust among the participants. This established relationship was also helpful in the recruitment of diverse participants for FGD and IDIs. The FGDs and IDIs were conducted in the native languages of participants, which ensured their active participation in the study. However, this study only explores the perspective of Bangladeshi and Nepalese communities living in Sydney; therefore, the study findings may not be generalisable to the broader Bangladeshi and Nepalese population in Australia, particualrly those living in rural and regional areas or to other South Asian migrant groups with different contextual experiences. We were unable not conduct IDIs face‐to‐face, which might limit richness in data that could have been achieved by in‐person interviews. Moreover, Bangladeshi and Nepalese communities are internally diverse, and we could not capture the intrasubgroup variations within these populations. This also highlights the importance of considering the heterogeneity of communities in designing preventive care services.

## 5 Conclusion

This qualitative study sheds light on the multilevel barriers and facilitators to accessing preventive care services among Bangladeshi and Nepalese communities in Sydney. Several barriers were identified across multiple levels, including cultural and religious perceptions, limited health literacy and lack of awareness of available preventive services, limited English language proficiency, insufficient culturally and linguistically tailored support, community‐level stigma and gaps in preventive programs. Conversely, facilitators included self‐focus on health, peer support, the cultural and linguistic competence of service providers, and the use of digital and social media for dissemination of inforamtion. The study highlights the importance of implementing multilevel, culturally appropriate and community‐based interventions to improve access to preventive care services among these underserved population groups. Furthermore, policymakers and public health practitioners should prioritise reframing existing policy frameworks and service delivery practices to ensure equitable access to preventive care services for Bangladeshi and Nepalese communities in Australia.

## Author Contributions

**Afsana Anwar:** conceptualisation, data curation, formal analysis, investigation, methodology, writing – original draft. **Grish Paudel:** conceptualisation, data curation, formal analysis, funding acquisition, writing – original draft. **Uday Narayan Yadav:** conceptualisation, investigation, methodology, funding acquisition, supervision, validation, writing – review and editing. **Md Nazmul Huda:** conceptualisation, data curation, investigation, funding acquisition, methodology, validation, writing – review and editing. **Abdullah Al Masud:** data curation, investigation, funding acquisition, validation, writing – review and editing. **Grace McKeon:** data curation, investigation, funding acquisition, validation, writing – review and editing. **Cathy O'Callaghan:** data curation, investigation, validation, writing – review and editing. **Ben Harris‐Roxas:** data curation, investigation, funding acquisition, validation, writing – review and editing. **Simon Rosenbaum:** conceptualisation, data curation, investigation, funding acquisition, methodology, supervision, validation, writing – review and editing. **Sabuj Kanti Mistry:** conceptualisation, investigation, methodology, data curation, formal analysis, funding acquisition, supervision, validation, writing – review and editing.

## Ethics Statement

The study was conducted in accordance with the Declaration of Helsinki and was approved by the UNSW Human Research Ethics Committee (iRECS6554) in June 2024.

## Consent

Written informed consent was sought from the participants before data collection.

## Conflicts of Interest

The authors declare no conflicts of interest.

## Supporting information

Supporting file 1.

Supporting file 2.

Supporting file 3.

## Data Availability

The data will not be shared publicly considering the privacy and anonymity of the participants.
