# Supplementary material for: Barriers and Facilitators to Accessing Preventive Services for Chronic Diseases Among People From Bangladeshi and Nepalese Backgrounds Living in Sydney
Source: Health Expect. 2026 Mar 24;29(2):e70644. doi: 10.1111/hex.70644 (PMC13087431; doi:10.1111/hex.70644)
Supplement: Supplementary file 1 — Supporting file 1. [file HEX-29-e70644-s002.docx]

**Supplementary file 1: Topic guides for participants**

***Focus group discussion guide for participants***

1. Introduction

Thank you for agreeing to participate in this study. The study aims to explore the barriers and facilitators to accessing chronic disease treatment and preventive services among people of Bangladeshi origin living in Sydney and potential ways to address the barriers to accessing these services. Therefore, in the focus group discussion, we will seek your views on these.

2.Permission to record.

With your permission, I would like to audio-record the discussion. Is that OK?

*If yes, indicate that you will start the discussion and the recording*

*If no, a*re you happy for me to make written notes? If yes, start the focus group discussion.

3. Questions for focus group discussion

Section A: Sociodemographic characteristics

- Age of the participants
- Gender of the participants
- Residency status (permanent/temporary)
- How many years staying in Australia?
- How many people do you have in your family (household)?

Section B: Perspectives and awareness of chronic diseases

1. What kind of chronic diseases (chronic disease is any disease that lasts for three months or more i.e., diabetes, heart diseases, kidney diseases) you see among the people of Bangladeshi origin living in Sydney?
2. Are you aware of the available preventive services for chronic disease (i.e., cancer screening, diabetes education group, physical activity programs, housing etc.) they access? Please explain.
3. What is the source of this information (i.e., social media, social gathering etc.)? Please elaborate.
4. What is your understanding of the term ‘physical activity’ in your own language? Please elaborate.

Section C: Barriers to accessing treatment and preventive services

1. Do you/your family/community members usually access preventive services (i.e., physical activity group, dietician) for chronic disease? Please elaborate.
2. To your knowledge, what measures are usually taken by people of Bangladeshi origin living in Sydney to treat chronic diseases (hints: GP, specialist, consulting neighbors, over the counter medicine, getting medicines from overseas, using WhatsApp’s to gather information, talking to a GP in Bangladesh via mobile etc.)?
3. Do you/your family/community members face any barriers/challenges in accessing chronic disease treatment and/or preventive services?
4. If yes, please discuss them. (Hints: language barriers, limited health literacy, finance/out-of-pocket cost, distance to health services, limited consultation time, patient-GP poor communication, language specific health providers, culturally inappropriate services, discrimination, lack of information etc.).
5. Do you think of any social factors as barriers to accessing chronic disease treatment and/or preventive services among Bangladeshi people living in Sydney? Please discuss the factors.
6. Are you/your family/community members able to understand healthcare providers’ (GP, nurses, midwives) suggestions always? Please discuss.
7. Do you think socio-economic status of people of Bangladeshi origin living in Sydney prevent them from seeking health care services such as treatment, diagnosis, accessing medications etc.? please discuss. Have you seen them refraining from seeking health care services due to these reasons? Is there any influence of visa status/health insurance coverage in accessing health services among them? Please discuss.
8. Are you/your family/community members able to afford healthcare services?
9. (If no), from diagnosis to care process, do you think they receive adequate treatment and/or preventive services as needed? Please discuss.
10. In your knowledge, what are the strategies followed by people of Bangladeshi origin living in Sydney to access treatment and/or preventive services for chronic disease management? Please elaborate these strategies.

Section D: Facilitators for accessing services

1. Have you seen people of Bangladeshi origin living in Sydney received support from their community members to access treatment and/or preventive services for chronic disease management? Please discuss in detail (these services). Has anyone ever come to you for this kind of support? In such event(s), what kind of support(s) did you provide? Please elaborate.
2. Do you/your family/community members prefer to be accompanied by someone from the Bangladeshi community when accessing services for chronic disease treatment and/or prevention? If yes, what roles are played by the accompanied person?
3. What do you think could be the potential ways for improving access to treatment and/or preventive services for chronic disease?
4. How can Bangladeshi community help to address barriers in accessing treatment and/or preventive services for chronic diseases (hint: information, navigation, accompany)? As a representative of your community, what roles can be played by you?
5. What are your recommendations to improve interaction within your community regarding accessing treatment and/or preventive services for chronic disease?

4. Closure of focus group discussion

That is all our question. Thanks for your time today. Do you have any questions for us?

***Interview guide for participants***

1. Introduction

Thank you for agreeing to participate in this study. The study aims to explore the barriers and facilitators to accessing chronic disease treatment and preventive services among people of Bangladeshi origin living in Sydney and potential ways to address the barriers to accessing these services. Therefore, in the interview, we will try to know about you/your family members’ perspectives of chronic disease management.

2. Permission to record.

With your permission, I would like to audio-record the interview. Is that OK?

*If yes, indicate that you will start the interview and the recording and proceed to the interview questions.*

*If no, a*re you happy for me to make written notes? If yes, start the interview

3. Interview Questions

Section A: Sociodemographic characteristics

- Age of the participants
- Gender of the participants
- Residency status (permanent/temporary)
- How many years staying in Australia?
- How many people do you have in your family (household)?

Section B: Perspectives and awareness of chronic diseases

1. Do you/your family members suffering/suffered from any chronic disease? (Chronic disease is any disease that lasts for three months or more e.g. diabetes, heart disease, kidney disease etc.)
2. Are you aware of the available preventive services for chronic disease i.e., cancer screening, diabetes education group, physical activity programs, housing etc.? Please elaborate.
3. What is the source of this information (i.e., social media, social gathering etc.)? Please elaborate.
4. What is your understanding of the term ‘physical activity’ in your own language? Please elaborate.

Section C: Barriers to accessing treatment and preventive services

1. Do you/family members usually access preventive services for chronic disease? Please elaborate your experience.
2. What measures are usually taken by you/your family members to treat chronic diseases (hints: GP, specialist, consulting neighbors, over-the-counter medicine, getting medicines from overseas, using WhatsApp’s to gather information, talking to a GP in Bangladesh via mobile etc.)?
3. Do you/your family members face any barriers/challenges in accessing chronic disease treatment and/or preventive services?
4. If yes, please discuss them. (Hints: language barriers, limited health literacy, finance/out-of-pocket cost, distance to health services, limited consultation time, patient-GP poor communication, limited language-specific health providers, culturally inappropriate services, discrimination, lack of information etc.).
5. Do you consider any social factors as barriers to accessing chronic disease treatment and/or preventive services? Please discuss barriers you or your family members have experienced, or you saw someone (Bangladeshi people living in Sydney) experienced it.
6. Are you/your family members able to understand healthcare providers’ (GP, nurses, midwives) suggestions always? Please discuss.
7. The socio-economic status of you/your family members ever prevented you/your family members from seeking healthcare services such as treatment, diagnosis, accessing medications etc.? Please discuss if you refrained from seeking health care services due to these reasons. Is there any influence of visa status/ health insurance coverage on you/your family members in accessing health services? Please discuss.
8. Are you/your family members able to afford healthcare services?
9. (If no), do you think you receive adequate treatment and/or preventive services as needed from diagnosis to care process? Please discuss.
10. In your knowledge, what are the strategies followed by people of Bangladeshi origin living in Sydney to access treatment and/or preventive services for chronic disease management? Please elaborate on these strategies.

Section D: Facilitators for accessing services

1. Have you or your household members ever received support from people of Bangladeshi origin living in Sydney to access treatment and/or preventive services for chronic disease management? Please discuss (these services). Has anyone ever come to you for this kind of support? In such event(s), what kind of support(s) did you provide? Please elaborate.
2. Do you/your family members are accompanied by someone from the Bangladeshi community (living in Sydney) when accessing services for chronic disease treatment and/or prevention? If yes, what roles are played by the accompanied person? Please elaborate.
3. According to you, what is the potential ways to improve access to treatment and/or preventive services for chronic disease?
4. How can the Bangladeshi community help to address barriers in accessing treatment and/or preventive services for chronic diseases (hint: information, navigation, accompany)? As a representative of your community, what roles can be played by you?
5. What are your recommendations to improve interaction within your community regarding accessing treatment and/or preventive services for chronic disease?

4. Interview Closure

That is all our question. Thanks for your time today.  Do you have any questions for us?
