# Supplementary material for: Barriers and Facilitators to Accessing Preventive Services for Chronic Diseases Among People From Bangladeshi and Nepalese Backgrounds Living in Sydney
Source: Health Expect. 2026 Mar 24;29(2):e70644. doi: 10.1111/hex.70644 (PMC13087431; doi:10.1111/hex.70644)
Supplement: Supplementary file 2 — Supporting file 2. [file HEX-29-e70644-s001.docx]

**Supplementary file 2: Barriers to access preventive care services with representative quotes**

| **Barriers to access preventive care services** | **Exemplar quotes of participants in FGDs and interviews** |
| --- | --- |
| **Individual-level barriers** | |
| Cultural and religious perceptions related to chronic disease | *1.1 “Especially many of us think that diabetes is God given. I have nothing to do here. But many people don't think that it is a modifiable matter, within a person's control. So, according to religious faith, it is considered pre-destination by all.”* **BD-FGD2-P3**  *1.2 “Culture is the biggest barrier among these. For all this, we cannot know anything. Added to that are our own health beliefs, taboos, our food habits.* **BD-FGD2-P3**  *1.3 “Sometimes, cultural background and strict rules create certain boundaries. For instance, some people feel uncomfortable opening to doctors, which can be a significant issue. This reluctance to speak openly about health concerns can prevent individuals from seeking help or accessing necessary healthcare services*.” **Nepalese-FGD2-P1** |
| Limited health literacy about chronic disease | *2.1 “I believe poor health literacy is one of the barriers. Even when people are diagnosed with chronic conditions, they know little about their health problems.”* **Nepalese-IDI-2**  *2.2 “I googled the chronic disease today at the time of interviewing you. I thought chronic disease meant cancer or something big like that. But I did not know that diabetes or hypertension are chronic diseases.”* **BD-IDI-3** |
| Lack of awareness about preventive services | 3.1 “*However, there appears to be a lack of awareness or understanding of what is available.”* **Nepalese-FGD3-P5**  *3.2 “No, we have not accessed any preventive services for managing my mother’s chronic conditions. We don’t know what services are available here in Australia or what the government offers.”* **Nepalese-IDI-9**  *3.3 “No, I do not know such services, and I have never accessed them. I believe this lack of awareness is not just limited to me but extends to most of the Nepalese community. Many Nepalese immigrants in Sydney are unaware of the preventive services available for chronic conditions.”* **Nepalese-IDI-6**  *3.4 “No. We have not heard anything, or we have never been told anything about preventive services.”* **BD-IDI-4** |
| Limited focus to self-care | *4.1 “Often, their priorities are focused more on work and financial stability than on health, which leads to low engagement in preventive healthcare.”* **Nepalese-FGD2-P1**  *4.2 “The pressure of balancing work, family life, and other responsibilities often leads to neglecting health until it becomes a serious issue. Many individuals get caught up in the daily grind and forget to prioritize their well-being.”* **Nepalese-FGD3-P3**  *4.3 “They neglect their health. Women are special. They completely ignore their health. I feel very sorry for the highly educated Bangladeshi sisters here. They come here and become so busy with their husband's career, job, and their children's school. They don't have time to make a phone call. Their confidence level has dropped so much. They do not exist on their own. As a result, various diseases occur. Diabetes is there, along with many other complex chronic diseases. Many also develop mental health-related problems.”* **BD-IDI-5**  *5.1 “The biggest problem in the Australian context is that we can't afford the time. This is a big problem.”*  **BD-FGD2-P3**  *5.2 “Another reason here is that in our daily life, we ​​all are too busy for work. It is not possible to find the rest of the time apart from work. We work here for 5-6 days*.” **BD-FGD3-P4**  *5.3 “Many get so caught up in their work or study life that their health often becomes a secondary priority. They focus on meeting deadlines, paying bills, and fulfilling other responsibilities, leaving little time to engage with health campaigns or activity programs that are available.”*  **Nepalese-FGD3-P5** |
| Fear of impact on visa status | *6.1 “Many people think that if I have any such health issue, then it will be a problem for getting my residency.”*  **BD-FGD1-P11**  *6.2 “I have seen closely, one of my friends, who didn't have permanent residency at the time, may have felt restricted in accessing healthcare services here.”* **Nepalese-FGD2-P3** |
|  | **Interpersonal level barriers** |
| Language barrier | *7.1 “Even the free-of-cost services that the government offers through community services often go untapped within the Nepali community. Whether knowingly or unknowingly, the language barrier prevents people from fully accessing these services*.” **Nepalese-FGD2-P1**  *7.2 “I didn't even understand their accent. Canterbury Hospital had an Indian Bengali-speaking interpreter in the antenatal sector; she explained everything to me. But unfortunately, I could not catch the classes on diabetes for even one day*.” **BD-IDI-5** |
| Limited availability of multilingual health education material and interpreter support | *8.1 “There is an interpreter, but there is no Bengali version for various documents. Many leaflets, but if they are in Bengali, people can read and understand. A lot of information can be found.”*  **BD-FGD2-P1**  *8.2 “Community language does not include Bengali*.” **BD-FGD2-P3**  8.3 “Like, sometimes we go to Bankstown, interpreters are available. Again, when I went to Canterbury, no interpreter was available.” **BD-FGD3-P4** |
| Inadequate cultural understanding among health care service providers | *9.1 “I also feel that GPs do not want to understand or assess our health beliefs, culture, and traditions.”* **Neplese-IDI-3**  *9.2 “Yes, I’ve heard from people about GPs making assumptions based on their race, and the person felt really bad about it.´* **Nepalese-IDI-10** |
| **Institutional-level barrier** | |
| Limited culturally and linguistically tailored navigation support and information access | *10.1 “There's a lack of information and navigating the healthcare system can be overwhelming without proper guidance or awareness. This leads to many people missing out on preventive care and timely treatment.”*  **Nepalese-FGD3-P5**  *10.2 “We are not familiar with the health systems or the available services for chronic conditions that are offered by the Australian healthcare system.”*  **Nepalese-IDI-8** |
| Indirect and/or infrequent public transport to health facilities | *11.1 “There are no buses in many places, which is also a problem.”*  **BD-FGD1-P5**  *11.2 “Like Canterbury Hospital, it is near, not too far. But leaving Lakemba, I think, becomes difficult. Because I must come to the station from my house, from here again go to Canterbury by train, then go again by bus.”* **BD-FGD1-P6**  *11.3 “They may not be able to drive, so how can they get there? There is no train or bus, maybe to that place, they don't want to go.”*  **BD-IDI-5** |
| Lack of after-hours preventive program/services | *12.0 “Various awareness sessions are held for diabetes. I do participate in those awareness sessions. But he doesn't have time as he has work at that time and the sessions are in working hours.”*  **BD-IDI-9** |
| **Community-level barriers** | |
| Community attitude towards health | *13.1 “Overall, Nepalese-origin individuals tend to neglect their health, skipping routine checkups and only seeking care during emergencies or severe illnesses*.” **Nepalese-IDI-5**  *13.2 “We (Nepalese) also have a belief that we will recover just by seeing a doctor. This belief is a major barrier in the prevention or management of chronic conditions.”*  **Nepalese-IDI-3**  *13.3 “The main problem of Bangladeshis is that they don't want to go to the doctor easily. They ignore health and well-being.”*  **BD-IDI-5** |
| Distance to health care facilities | *14.0 “Distance to healthcare facilities also plays a role in seeking care. In the past, I had to travel a long distance for counseling, which was difficult for me.”* **Nepalese-IDI-2** |
| Limited connection and engagement within the community | *15.1 “Here's what I see, you just be yourself, no one tells you anything. Everyone is busy with themselves.”* **BD-IDI-1**  *15.2 “Additionally, social interactions and community involvement tend to be minimal, as many are focused on their individual responsibilities. Participation in community and volunteering activities is quite low, further limiting opportunities for social connection and engagement.”* **Nepalese-FGD2-P1** |
| Stigma associated with chronic disease | *16.1 “It's a big barrier to going to the doctor plus a barrier to sharing information. When I run any session in the community, there are many aunts, uncles or aunties who come to me and say they have something to talk about. It may take them a long time to think about sharing this topic. This attitude is strong among Bangladeshis.”* **BD-IDI-5**  *16.2 “I am not sure about other communities, but social stigma plays a significant role in the Nepalese community. Many individuals with mental health conditions prefer to remain unidentified and isolate themselves due to fear of being judged by their families, relatives, or community. This hesitation prevents them from seeking timely medical support.”* **Nepalese-IDI-6**  *16.3 “The social factors play a big role in creating barriers within the Nepali community. There's a strong cultural tendency to care about how others perceive you, especially when it comes to health issues like heart disease, cancer, or sexually transmitted diseases. People often feel a sense of shame or fear of judgment, so they keep it to themselves instead of seeking help or sharing their struggles. This stigma can prevent many from accessing the care they need, and it’s something that could be addressed by fostering a more open and supportive environment where seeking help is seen as a sign of strength, not weakness.”* **Nepalese-FGD3-P6** |
| **Policy-level barriers** | |
| Inadequate coverage of preventive services | *17.1 “The accessibility of these programs is not user-friendly, nor are they exposed*.” **BD-FGD2-P3**  *17.2 “These programs are not visible. Not particularly visible in community settings, and they lack accessibility. The main reason for the lack of accessibility is that they are not exposed. and not culturally acceptable at the community level.”*  **BD-FGD2-P3**  *17.3 “Yes, there are free health education programs for diabetes management. However, the availability of these services can vary depending on the local council, authority, or GP.”*  **Nepalese-IDI-11** |
| Lack of patient-centric preventive care | *18.0 “These programs are not just patient-centered services in New South Wales; they are more advocates. They do more advocacy, but they don't do much for the common people.”*  **BD-FGD2-P3** |
