# Supplementary material for: Barriers and Facilitators to Accessing Preventive Services for Chronic Diseases Among People From Bangladeshi and Nepalese Backgrounds Living in Sydney
Source: Health Expect. 2026 Mar 24;29(2):e70644. doi: 10.1111/hex.70644 (PMC13087431; doi:10.1111/hex.70644)
Supplement: Supplementary file 3 — Supporting file 3. [file HEX-29-e70644-s003.docx]

**Supplementary file 3: Facilitators to access preventive care services with representative quotes**

| **Facilitators to access preventive care services** | **Exemplar quotes of participants in FGDs and interviews** | |
| --- | --- | --- |
| **Individual-level facilitators** | | |
| Self-awareness and ownership of health | *1.0 “If my diabetes goes above 9, I feel very sick. I see many going on 12/13, but I can't. My body tells me that my diabetes has increased, so I am forced to do so much. That’s why I am so aware.”* **BD-IDI-6** | |
| Knowledge about available preventive services | *2.1 “As far as I know Metro Assist or the library sometimes conducts some sessions of yoga exercise, chair sitting type of activities for the elderly, long-term spine problems, diabetes problems. Such as exercise and morning walks which have refreshment activities, they do these. I have seen such activities. Chair sitting activity, morning walk activity, these I have seen on behalf of Metro Assist plus the initiatives taken by Lakemba Library in the community.” BD-FGD3-P4*  *2.2 “I know a program for girls’ breast cancer.” BD-FGD3-P5* | |
|  | **Interpersonal level facilitators** | |
| Cultural and linguistic competency of the healthcare service providers | *3.0 “I find it easier to communicate with Nepalese GPs as we share the same language and cultural background.”*  **Nepalese-IDI-1** | |
| Community-based peer support and informal information navigation | *4.1 “Besides, I think that there are marathons, walking, people go there and share personal things, who is doing what.” (Table 3, Quote 4.1,* **BD-IDI-5)**  *4.2“Social gatherings where people talk openly about chronic disease. There is a group of people who are diabetic. They will walk or do some activity together. What measurements are taken, I will share. I think when there is talk, sharing, then communication will be fine.” (Table 3, Quote 4.2,* **BD-IDI-7)**  *4.3 “As I work for a community organisation, many people I invite, like this sister. She is coming alone from New Town. 10 more people did not come. She is able to give the information that there is such an organization that works. These 10 people can give information to another10 people, like networking.”* **BD-FGD1-P11**  *4.4 “When someone in my family has a chronic condition, I usually seek advice from other senior Nepalese people or contact the hospital for information. This is what my family and I typically do.”* **Nepalese-FGD-P4**  *4.5 The main source of information is through friends or peers. In my area (Edmondson Park), there’s a Nepalese community chat group where we share information about these services. We also learn from friends verbally.”* **Nepalese-FGD-P1**  *4.6 “I tell people what I know when I go to a program or go for a walk. In this way, it is better to do these things.”* **BD-IDI-8**  *4.7 “I found people helpful here. Specially those who have been in Australia for many years. Those who are in the initial stages do not know much. But those who have been there for many years, people give advice if someone go to them for advice.”* **BD-IDI-1**  *4.8 “If anyone knows any programs. or benefit from treatment somewhere, he can tell others.”* **BD-IDI-7**  *4.9 “I have shared my experiences with others regarding my health conditions and management strategies. Some people dealing with mental health issues have reached out to me, and I have tried to encourage and motivate them.”* **Nepalese-IDI-1** | |
| **Institutional-level facilitators** | | |
| Availability of multilingual health education resources and interpreter support | *5.1 “There is an interpreter, but there is no Bengali for various documents. Many leaflets, but if they are in Bengali, people can read and understand. A lot of information can be found.”*  BD**-FGD2-P1**  *5.2 “Since the GP is the first point of contact here, the instructions can be written down to him in Bengali. In the medical centers that we have, it may be written there, what extra support is there for chronic conditions.”*  BD**-FGD-3** | |
| Digital and social media as source of information | *6.1 “ I believe social media is the most practical solution for spreading information, as it would be challenging to visit every household individually. Platforms like Facebook or TikTok offer an efficient way to reach a large audience quickly. By creating dedicated health-focused pages, we can share valuable information, tips, and resources directly with the community.”* Nepalese**-FGD-P3**  *6.2 “Yes, social media is so common among almost all the people. It would be effective if some health-related awareness are shared through the social media through different Nepali community pages would improve the awareness level.”*  Nepalese**-FGD-P5**  *6.3 “Yes, any information related to chronic disease can be shared or asked for in Facebook and WhatsApp group.”* **BD-IDI-5**  *6.4 “ I think because everyone uses mobile. And there are many of us who are not used to email. We may see a lot of things on the internet or email. But those with chronic diseases are generally 40 plus or 35 plus in age. For them mobile SMS can be given in different languages, it can be helpful to pass information like we get for different things.”*  **BD-FGD3-P4**  *6.5 “Radio ads could be an effective way to raise awareness about preventive health services and available healthcare programs. Many people still listen to the radio and such ads would catch the attention of those who may not actively seek out information online or through pamphlets.”* **Nepalese-FGD-P6** | |
| **Community-level facilitators** | | |
| Community-led psychosocial support | *7.0 “The big thing about chronic disease is that when people know that they have a chronic disease, and it will slowly make them sick, there is no way to survive, so mental support is very important. In this case, Bangladeshis can give each other mental support. They can help in different ways. The help that I get. Many Bangladeshi brothers whom I don't even know visit me.”* ***BD-IDI-11*** | |
| Reorientation and utilisation of existing community organisations for preventive care | *8.1 “However, I believe these organizations tend to be more reactive than proactive. There is a need for them to focus more on raising awareness and actively advocating for health issues at the community level, rather than only responding to situations as they arise.”* Nepalese**-FGD2-P3**  *8.2 “Also, the Nepali welfare community or organisation should also consider health as a priority area and should work in this area with high priority to help and support either by awareness raising or by acting as a navigator or through some financial assistance.”* **Nepalese-FGD2-P5**  8.3 “These organizations are primarily involved in fundraising for individuals with major illnesses or to send deceased individuals' bodies back to Nepal. It seems their main focus is to support the repatriation of bodies. I haven’t heard or seen any preventive programs or services being organized by them.” Nepalese-IDI-3  8.4 “From what I know, they are active in organizing social events like cultural programs during festivals. Sometimes, they raise funds to help people who are critically ill or for sending bodies back to Nepal. However, I haven’t seen much focus on health education or related issues.” Nepalese-IDI-9  8.5 “I don't see Nepali organizations contributing significantly to the welfare of the Nepali community when it comes to preventive services for chronic disease management. While some community organizations may focus on cultural events and politics, there seems to be a lack of emphasis on health-related education or services that can help prevent or manage chronic diseases within the community.” Nepalese-IDI-10  8.6 “I think it would be better if Nepalese organizations could shift their focus from politics to more social aspects. Right now, they mostly work on a demand basis, but it would be more beneficial if they took the initiative to organize informational campaigns and preventive services for the community.” Nepalese-IDI-10 | |
|  | *8.7 “Some organizations help navigate services for people with mental health issues, connect individuals with the right healthcare providers, assist people who need to return to Nepal due to health conditions, and help families come to Australia to care for sick relatives.” Nepalese-FGD1-P4*  *8.8 “If there are any doctors within the NRNA members, they can initiate these programs. They should identify existing services and develop and distribute brochures within all Nepalese communities.” Nepalese-FGD1-P2*  *8.9 “Additionally, collaborating with these organizations could create opportunities for more personalized outreach, allowing for culturally sensitive discussions around preventive care and health services.” Nepalese-FGD2-P1*  *8.10 “Nepalese welfare organizations are primarily focused on hosting entertainment programs, like concerts and festivals with Nepalese singers and artists. They don’t seem to prioritize health-related programs. These organizations should establish a network to distribute health bulletins with information on available services for the prevention and management of chronic diseases within the Nepalese community.” Nepalese-IDI-3* | |
| Utilisation of existing community gatherings as a point of preventive care outreach | *9.1 “The Bangladeshi community has many festivals. There are various health promotion programs in these festivals, those who work with the community can provide booths. They can do community consultation within the booth. If you go to these places, you will get this service.” BD-FGD2-P3*  *9.2 “The biggest thing is that we all go to school, go to mosque, go to work, go to the GP. Everyone is a part of the community. In this way, communication can be done within the community.” BD-FGD3-P4*  *9.3 “Awareness sessions should also be held during Nepalese events, festivals, and other community programs.” Nepalese-FGD1-P6* | |
| **Policy-level facilitators** | | |
| Integrated service provision for immigrants | | *10.0 “Immigrants face multiple challenges as they move from one country to another. He will not go to that country again. At the time of coming from the country, the property is being sold. So, here, comprehensive patient-centered needs for immigrants are need to be understood by the government. And these should be tagged with support services like Centrelink, MediCare.”*  **BD-FGD1-P3** |
